# Heart Rate Variability Monitoring Based on Doppler Radar Using Deep Learning

**DOI:** 10.3390/s24072026

**Published:** 2024-03-22

**Authors:** Sha Yuan, Shaocan Fan, Zhenmiao Deng, Pingping Pan

**Affiliations:** School of Electronics and Communication Engineering, Shenzhen Campus of Sun Yat-sen University, Shenzhen 518107, China; yuansh23@mail2.sysu.edu.cn (S.Y.); fanshc3@mail2.sysu.edu.cn (S.F.); panpp3@mail3.sysu.edu.cn (P.P.)

**Keywords:** HRV, radar, deep learning, neural network

## Abstract

The potential of microwave Doppler radar in non-contact vital sign detection is significant; however, prevailing radar-based heart rate (HR) and heart rate variability (HRV) monitoring technologies often necessitate data lengths surpassing 10 s, leading to increased detection latency and inaccurate HRV estimates. To address this problem, this paper introduces a novel network integrating a frequency representation module and a residual in residual module for the precise estimation and tracking of HR from concise time series, followed by HRV monitoring. The network adeptly transforms radar signals from the time domain to the frequency domain, yielding high-resolution spectrum representation within specified frequency intervals. This significantly reduces latency and improves HRV estimation accuracy by using data that are only 4 s in length. This study uses simulation data, Frequency-Modulated Continuous-Wave radar-measured data, and Continuous-Wave radar data to validate the model. Experimental results show that despite the shortened data length, the average heart rate measurement accuracy of the algorithm remains above 95% with no loss of estimation accuracy. This study contributes an efficient heart rate variability estimation algorithm to the domain of non-contact vital sign detection, offering significant practical application value.

## 1. Introduction

Heart rate variability (HRV) refers to the fluctuation in cycle-to-cycle intervals of heart rate or the pace of cardiac pulsations. HRV holds diverse clinical implications, particularly in conditions like coronary heart disease [1], hypertension [2], and diabetic neuropathy, which manifest as neurological impairments [3] in the cardiac system. Moreover, HRV serves as a valuable tool for diagnosing different types of arrhythmias [4,5,6], guiding pharmacological interventions and effectiveness judgment [7]. As a non-invasive physiological indicator, HRV has attracted significant attention, showcasing its potential for application in clinical settings.

Currently, the analysis of HRV involves the examination of the time series of heartbeats and intervals between heartbeats obtained through electrocardiography (ECG) or pulse measurements [8,9,10]. However, these methodologies necessitate physical contact, presenting certain drawbacks. The physical contact required for these measurements may induce discomfort in users and impose limitations on the applicable contexts. Prolonged wearing of sensors to monitor respiratory and cardiac signals can result in discomfort for patients over extended periods, potentially affecting their overall well-being. Moreover, contact-based measurements prove impractical for certain patient populations, including those with conditions such as burns, infectious diseases, and mental health issues, as well as in scenarios like personal healthcare and battlefield rescue. As a solution to these challenges, the pursuit of non-contact measurements has emerged as a highly desirable objective. Various endeavors have been undertaken to explore the use of radar technology for non-contact vital sign measurements [11,12,13].

In the case of an orthogonal receiver radar with I/Q channels, the phase can be obtained by applying arctangent demodulation [14,15] or the extended DACM algorithm [16,17]. Mehrdad Nosrati and Negar Tavassolian [18] introduced a model based on a Gaussian pulse sequence and employed the frequency–time phase regression (FTPR) algorithm for heart rate estimation. Doppler radar is utilized to acquire the HRV index. Nonetheless, the computation of HRV relies on a signal duration of 10 s, imposing a constraint on its capacity to effectively capture rapid fluctuations in heart rate within brief temporal intervals.

Due to the large variation in heartbeat waveforms, Sakamoto et al. [12] claimed that methods based on Fourier transform and periodicity may not be reliable enough in estimating instantaneous heart rate (HR). They have introduced six artificial waveform features along with a feature topological signal for identifying unreliable feature points. Nevertheless, the identified feature points exhibit a significant reliance on the waveform, posing challenges in the accurate extraction of these points from the waveform data. Additionally, the susceptibility of radar signals to interference from noise and clutter further complicates the task of extracting feature points, adding an additional layer of difficulty to the process.

Based on the new Ka-band Doppler radar structure, Hosseini and Amindavar [19] proposed a filter with time-varying coefficients to detect HRV without tracking HR. This method is based on a specific radar architecture and requires further verification before it can be applied to mainstream radar architectures. Notably, studies revealed substantial differences in chest wall displacement between breathing and a heartbeat, with breathing inducing a 4–12 mm [20,21] body surface displacement and a heartbeat causing less than a 0.6 mm [22] displacement. In the relaxed state of normal breathing, the energy of the respiratory signal exceeds the energy of the heartbeat signal. The respiratory signal almost masks the heartbeat signal. Separating heartbeat signals from mixed signals is a challenging task.

Moreover, HR estimation faces significant interference from respiratory harmonics and intermodulation terms between breathing and a heartbeat. Various studies have proposed solutions to address this challenge. In [23], the authors select components within the second harmonic frequency band of the heartbeat spectrum to directly reconstruct the second harmonic signal. On the other hand, ref. [24] focuses on suppressing harmonics and intermodulation terms to enhance the signal-to-interference ratio of the heartbeat signal. The differential enhancement (DE) method, introduced by Xiong et al. [25], targets the elimination of breathing harmonics, and its efficacy has been validated through rigorous mathematical analysis and experiments.

In recent years, deep learning technology has found widespread application in radar data processing. For instance, in article [26], human heart rate and activity are detected through multi-task transfer learning. Haili Wang et al. [27,28] utilize deep learning to reconstruct the heartbeat signal, achieving high-precision heart rate detection. It is noteworthy that the signal lengths used in most current heartbeat detection studies are 10 s or longer [29,30,31,32]. For instance, article [28] employs one minute of data. However, such prolonged signals may not adequately capture HRV.

This paper introduces a rapid heartbeat frequency estimation method leveraging deep learning. To facilitate frequency domain signal analysis, we devised a network architecture comprising a frequency representation module and a residual in residual module. This design effectively mitigates breathing interference, ensuring precise estimation of the heartbeat frequency. Notably, even with only 4 s of data, the estimation accuracy remains uncompromised. Extensive validation on simulation data, open-source datasets, and self-collected data demonstrates the method’s proficiency in monitoring HRV. Compared with other methods, our method has the following advantages:

1. Effective respiratory signal interference elimination: our method successfully mitigates the interference of respiratory signals, enhancing the accuracy of heartbeat frequency estimation.
2. High detection accuracy: the proposed method achieves high detection accuracy, as evidenced by an average heartbeat time interval error of less than 2%.
3. Short signal length: The signal length is notably brief, lasting only 4 s. This enables the tracking of dynamic changes in HR over short periods, facilitating continuous heart rate monitoring.
4. No specific prerequisites for the radar type: the proposed method can be directly applied to data collected by various radar systems, including but not limited to Frequency-Modulated Continuous-Wave (FMCW) radar and Continuous-Wave (CW) radar.

The subsequent sections of this article are structured as follows: Section 2 describes data acquisition and related processing, providing a detailed introduction to the proposed method. Section 3 presents the experiments and their corresponding results. In Section 4, a comprehensive discussion and analysis of the introduced topics are provided, accompanied by relevant insights. Finally, Section 5 draws conclusions.

## 2. Materials and Methods

### 2.1. Signal Model

**Figure 1:**
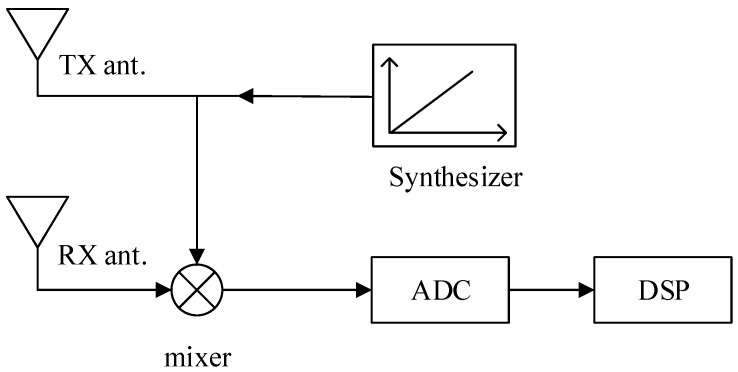
FMCW radar system block diagram. System includes transmit antennas, receive antennas, synthesizer, mixer, analog-to-digital converter, and digital signal processing elements.

The system framework of FMCW radar is shown in Figure 1. The signal synthesizer generates a chirp signal, which is radiated through the transmitting antenna. Upon interaction with a living organism, a portion of the electromagnetic wave is absorbed, while the remainder is reflected back and captured by the receiving antenna. The micro-motion of the chest wall caused by breathing and heartbeats is loaded in the echo signal. The signal of the receiving antenna and the signal of the transmitting antenna are mixed to obtain an intermediate frequency (IF) signal. Following analog-to-digital conversion by the analog-to-digital converter (ADC), the resulting signal is forwarded to the digital signal processor (DSP) for subsequent algorithmic processing.

Equation (1) gives the form of the transmitting signal:

(1) $$s_{T}\left(t\right)=A_{T}\exp\left(j2\pi \left(f_{0}t+\frac{1}{2}Kt^{2}\right)+\phi_{0}\right).$$

where $A_{T}$ denotes the amplitude of the transmitted signal and $f_{0}$ is the frequency at the initial moment of signal transmission. *K* represents the frequency modulation slope, $K=B/T_{c}$, $T_{c}$ is the frequency sweep period, and $\phi_{0}$ is the initial phase. The received signals are expressed in Equation (2).

(2) $$s_{R}\left(t\right)=A_{R}\exp\left(j2\pi f_{c}\left(t-\tau \right)+j\pi K{\left(t-\tau \right)}^{2}+\phi_{0}\right).$$

where $A_{R}$ denotes the amplitude of the transmitted signal, and τ=2R/c represents the time delay between the transmitted and received signals. The variable *R* represents the radial distance from the target to the radar, and *c* represents the speed of light. Under the stop-and-go approximation, the human target and the radar remain relatively stationary during the fast time. The small change in *R* is determined by the body surface caused by breathing and a heartbeat, and *R* can be modeled as Equation (3).

(3) $$R=R_{0}+x\left(t\right).$$

The IF signal is the result of mixing the received signal and the local oscillator signal, and the local oscillator signal can be regarded as a copy of the transmitted signal. The working process of the mixer can be understood in this way. First, the received signal is multiplied by the local oscillator signal, and then the high-frequency components are filtered out through a low-pass filter, and the low-frequency components are retained. The frequency mixing is carried out twice, namely the mixing of the received signal and the local oscillator signal, and the mixing of the received signal and the signal after the phase shift of the local oscillator by 90 degrees. This will form the in-phase component and the quadrature component of the baseband signal (also called the IF signal). The I/Q two-way IF signal is expressed in complex form as Equation (4).

(4) $$y\left(t\right)=A_{IF}e^{j\phi_{IF}\left(t\right)}.$$

where the I and Q signals are $R_{I}\left(t\right)=\sin\left(\phi_{IF}\left(t\right)\right)$ and $R_{Q}\left(t\right)=\cos\left(\phi_{IF}\left(t\right)\right)$, respectively. Perform arctangent demodulation [14,15] on the radar received signal to obtain the phase of the IF signal, as shown in Equation (5).

(5) $$\phi_{IF}\left(t\right)=\arctan\left(\frac{R_{I}\left(t\right)}{R_{Q}\left(t\right)}\right).$$

According to the derivation and ignoring some items with relatively small values, the expression of the IF signal can finally be obtained, as shown in Equation (6).

(6) $$\phi_{IF}\left(t\right)=\frac{4\pi R}{\lambda }.$$

where λ is the wavelength of the carrier signal. It can be seen that the phase of the IF signal contains the breathing and heartbeat information, which is proportional to the chest wall displacement caused by the breathing and heartbeat. This makes it possible to use the phase of the IF signal to obtain the respiratory and heartbeat signals.

### 2.2. Mathematical Model of Chest Displacement

In most current studies, for the modeling and analysis of vital signals, the simulation of breathing and heartbeat signals is generally set as the superposition of two sinusoidal signals with different amplitudes and frequencies. However, this is not consistent with the actual situation. In fact, breathing and heartbeat signals are not single-frequency signals but contain harmonic components [33,34,35].

According to the mathematical model proposed in [36], the model only defines the breathing pattern as two repeated phases, namely the inhalation and exhalation phases. Inhalation and exhalation result in a change in lung volume with proportional chest wall motion. The chest wall displacement curve caused by breathing consists of an inspiratory parabolic curve and an exponential expiratory curve, expressed as Equation (7).

(7) $$x_{r}\left(t\right)=\left\{\begin{array}{cc} \frac{-A_{r}}{T_{i}\cdot T_{e}}\cdot t^{2}+\frac{A_{r}\cdot T_{r}}{T_{i}\cdot T_{e}}\cdot t & t\in \left[0,T_{i}\right]\\ \frac{A_{r}}{1-e^{-\frac{T_{e}}{\tau }}}\cdot \left(e^{-\frac{\left(t-T_{i}\right)}{\tau }}-e^{-\frac{T_{e}}{\tau }}\right) & t\in \left[T_{i},T_{r}\right] \end{array}\right.$$

where $\left[0,T_{i}\right]$ represents the inhalation phase, and $\left[T_{i},T_{r}\right]$ represents the exhalation phase, $T_{i}$ and $T_{e}$ represent the duration of the inspiratory and expiratory phases, respectively. $T_{r}$ represents the length of a respiratory cycle, and $A_{r}$ represents the maximum amplitude of respiration. The respiratory rate can be calculated as $f_{r}=2\pi /T_{r}$. τ is the time constant of the respiratory profile, which determines the rate of the filling and emptying of gas in the lungs. The displacement caused by heartbeat activity refers to the Gaussian pulse sequence model proposed in [18]. The Gaussian pulse expression is given by Equation (8).

(8) $$x_{\mathrm{guassian}\;}\left(t\right)=A_{h}e^{-\frac{{\left(t-T\right)}^{2}}{2\sigma^{2}}}.$$

where $A_{h}$ represents the maximum amplitude value of the heartbeat, *T* and σ are constant parameters. It is known that the time interval between two consecutive pulses of the ECG signal is not a constant value but changes with time. The heartbeat model can be expressed as Equation (9).

(9) $$x_{h}\left(t\right)=\sum_{n}A_{n}e^{-\frac{{\left(t-t_{n}\right)}^{2}}{2\sigma^{2}}},\;t_{n}=T_{1},T_{1}+T_{2},T_{1}+T_{2}+T_{3},\ldots .$$

where $T_{n}$ represents the time interval between the pulse *n* and the pulse n−1, and $A_{n}$ represents the amplitude of the *n*-th pulse. In the duration $\left[0,T_{n}\right]$, the average pulse time interval is $T_{h}=\frac{1}{n}\sum_{i=1}^{n}T_{i}$, and the heartbeat frequency is $f_{h}=2\pi /T_{h}$. In the case of no random body movement, that is, when the person is still, the displacement of the chest surface (as shown in Equation (10)) measured by the radar system at time *t* is the superposition of the vibration caused by the heartbeat and the breathing movement of the thoracic cavity.

(10) $$x\left(t\right)=x_{r}\left(t\right)+x_{h}\left(t\right).$$

### 2.3. Data Acquisition and Preprocessing

**Figure 2:**
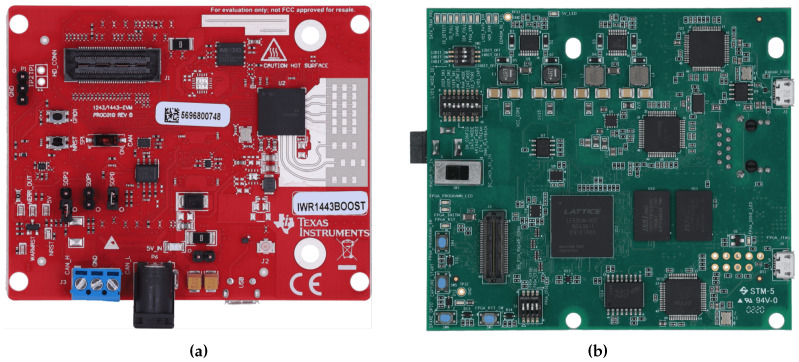
Radar vital sign detection and monitoring system hardware experiment platform. (**a**) IWR1443BOOST Evaluation Board. (**b**) The DCA1000 EVM.

**Figure 3:**
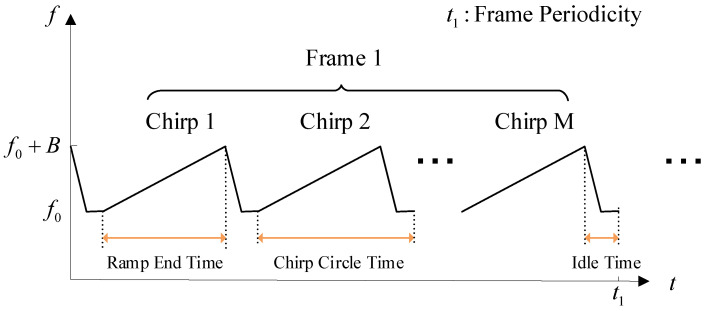
The instantaneous frequency representation of the signal transmitted by a radar system. The starting frequency is $f_{0}$, the bandwidth is *B*, and the chirp duration is composed of Ramp End Time and Idle Time.

The non-contact biological radar cardiopulmonary sign detection and monitoring system hardware experiment platform includes millimeter wave radar sensor IWR1443 Boost and DCA1000 EVM, as shown in Figure 2. These two devices are from the Texas Instruments Company of the United States, headquartered in Dallas, Texas. In this paper, IWR1443 is used to obtain analog-to-digital (AD) data in vital sign detection, and the binary data file of AD sampling is generated through the data acquisition board. Binary data files were preprocessed with Matlab. The RF front end of IWR1443 contains 3 transmitting antennas (T × 0, T × 1, T × 2) and 4 receiving antennas (R × 0, R × 1, R × 2, R × 3). The radar has the function of multi-target detection and positioning. In order to facilitate data processing, single-target vital sign detection uses 1 transmitting antenna (T × 0) and 4 receiving antennas (R × 0–R × 3), and only takes the data from one receiving antenna for algorithmic processing. The ADC data storage format is a 16-bit complex number format. The radar sensor parameter settings are shown in Table 1. The instantaneous frequency representation of the radar-transmitted signal is shown in Figure 3. A frame includes *M* chirps, and there are *N* frames in total.

**Figure 4:**
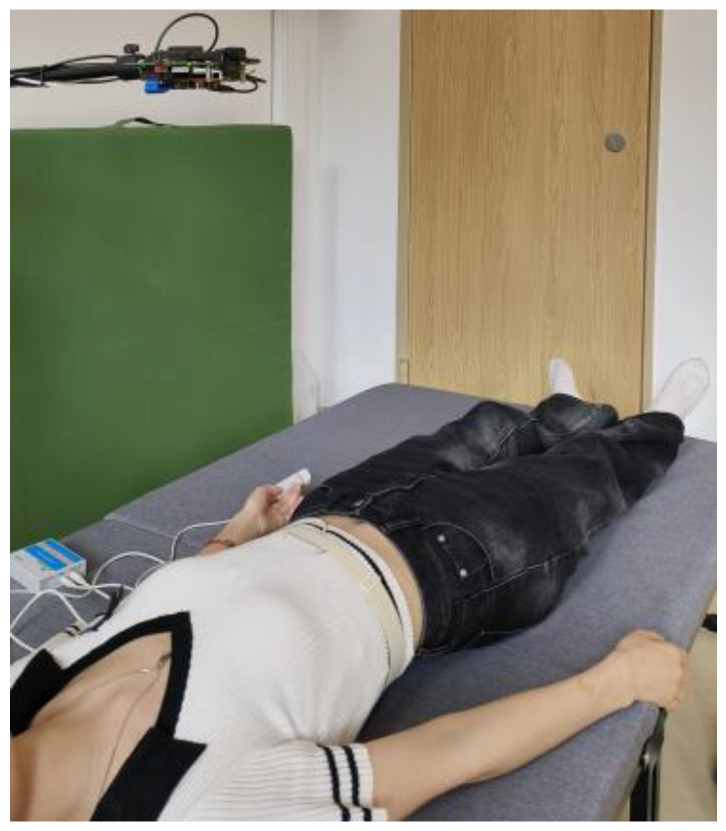
The experimental environment.

The experimental environment is shown in Figure 4. Participants were told to breathe freely and steadily as usual in a relaxed state during the trial, rather than very deep and excessive breathing. In addition, they were asked to avoid excessive body and hand movements, as rapid movements may mask small chest wall movements from heartbeats, thereby affecting detection.

The method of collecting vital sign signals through radar is mainly to measure the phase at the corresponding distance from the target. Combining the I and Q road data output by the radar, the obtained radar data can form a complex two-dimensional matrix. Each row in the two-dimensional matrix corresponds to a continuous sample of a pulse echo. According to the parameters in Table 1, there are five chirps in one frame, and 200 points are sampled in each chirp sweep cycle.

In this experiment, we take the first chirp signal of each frame to obtain the velocity dimension (also called slow time dimension) data. At this time, because the time is very short, it can be considered that the distance of the target has basically not changed (even for a moving target). Each column in the two-dimensional data matrix represents a series of pulse measurements of the same distance unit, that is, the distance dimension (also called fast time dimension) data obtained by sampling within a chirp frequency sweep period.

**Figure 5:**
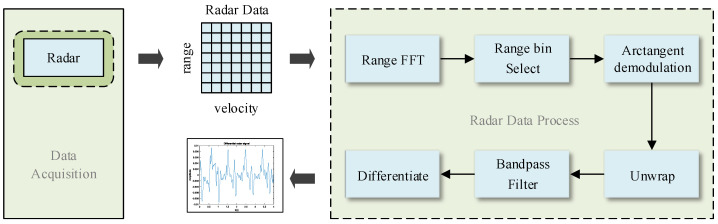
Flow chart of radar data preprocessing.

**Figure 6:**
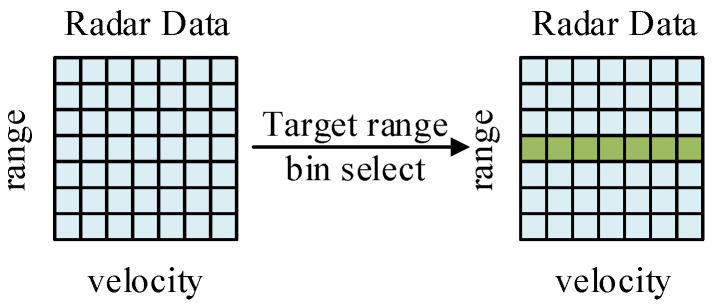
Determine the range bin where the target is located.

Figure 5 describes the radar signal and reference signal preprocessing flow. In order to obtain the target distance, the radar received signal is first processed by fast Fourier transform (FFT) in the fast time dimension, also called range-FFT or 1D−FFT. Then, within the effective distance range of the target, potential targets are locked by extracting the indexes corresponding to the first K maximum peaks in the 1D-FFT spectrum. Finally, the distance window where the target is located is determined through the algorithm, and a good superimposed signal of human breathing and the heartbeat is obtained, as shown in Figure 6.

According to Equation (5), the phase is extracted using the principle of the arctangent function. Usually, the thorax movement caused by human breathing is generally about 12 mm, which is several times the wavelength of radar (the wavelength of 77 GHz radar is about 4 mm). The amplitude range of the phase value obtained by the arctangent function is $\left[-\frac{\pi }{2},\frac{\pi }{2}\right]$, which causes the real phase value generated by the respiratory and heartbeat signal to be folded in the range of $\left[-\frac{\pi }{2},\frac{\pi }{2}\right]$. To obtain the real phase value of the radar signal, it is necessary to unwrap the obtained phase information. This method can correct the phase information and realize the removal of phase ambiguity.

The amplitude of the respiratory signal is several times or even dozens of times that of the heartbeat signal. In addition, the actual respiratory signals contain respiratory harmonics. When these harmonics are close in frequency to the heartbeat signal, it is easy for the algorithm to misidentify the breathing harmonic signal as a heartbeat signal. This is a great challenge to the detection of the heartbeat signal and causes great interference to the heartbeat estimation. Therefore, this paper uses a fourth-order Butterworth band-pass filter to filter the above-mentioned phase signal, and the cut-off frequencies are 0.67 Hz and 15 Hz, respectively. After filtering, a signal containing changes in the heart rate of the human target will be obtained. Alternatively, if it is assumed that the chest wall displacement consists of only two sine waves, the expression would be as shown in Equation (11).

(11) $$x\left(t\right)=A_{r}\sin\left(2\pi f_{r}t\right)+A_{h}\sin\left(2\pi f_{h}t\right).$$

where $A_{r}$ and $A_{h}$ represent the breathing amplitude and the heartbeat amplitude, respectively. $f_{r}$ and $f_{h}$ represent the respiratory rate and the heart rate, respectively. We calculate the first-order derivative of the chest wall displacement and obtain the differential signal as shown in Equation (12).

(12) $$\frac{dx(t)}{dt}=2\pi f_{r}A_{r}\cos\left(2\pi f_{r}t\right)+2\pi f_{h}A_{h}\cos\left(2\pi f_{h}t\right).$$

After differentiation, the amplitude of the heartbeat signal increases by $2\pi f_{r}$ times. Compared with the amplitude of the respiratory signal that increases by $2\pi f_{h}$ times, the heartbeat signal increases more, which can reduce the interference of breathing on the heartbeat. The heartbeat waveform recovered from the differentiated signal has obvious periodicity. Finally, the radar signal and the reference signal are downsampled so that their sampling frequencies are unified to the same value. Finally, the radar signal and the reference signal are downsampled so that their sampling frequencies are unified to the same value.

### 2.4. Proposed Network

**Figure 7:**
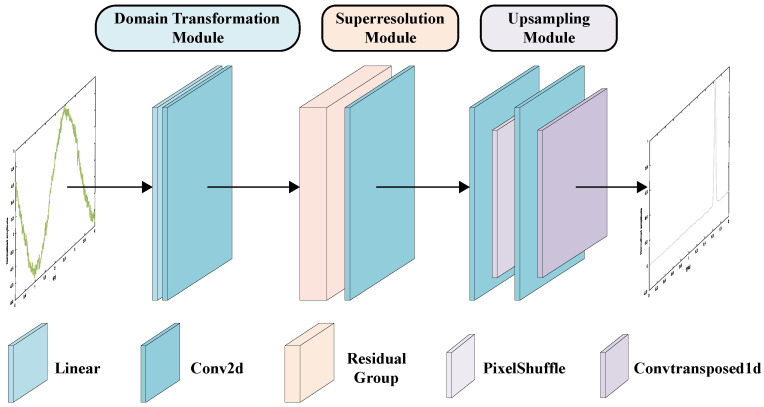
Overall network framework.

The proposed network framework includes a domain transformation module, a super-resolution module, and an upsampling module, as shown in Figure 7. The network model is introduced in detail below.

(1) Input layer: The input signal is the phase information of the signal acquired by the radar after preprocessing. By normalizing it, the difference caused by the signal amplitude values collected by different individuals and different time periods can be avoided. It is converted into a dimensionless pure value, and the data are uniformly mapped to the interval [0, 1], which can eliminate the unit limitation of the data. This can not only improve the convergence speed of the model but also the accuracy of the model. At the same time, it can ensure that the small value of the output data is not swallowed.

(2) Domain transform module: The domain transformation module consists of a fully connected layer and a 2D convolutional layer. The fully connected layer realizes the conversion from the time domain to the frequency domain, and then the 2D convolutional layer is used to realize the suppression of noise and harmonics. The following explains how the fully connected layer achieves frequency characterization. Ignoring the bias term, the fully connected layer can be represented as Equation (13).

(13) $$\begin{array}{cc} y & =xW=\sum_{n=0}^{N-1}x\left(n\right)\left(W_{R}+jW_{I}\right)\\ \; & =\sum_{n=0}^{N-1}x\left(n\right)W_{R}+j\sum_{n=0}^{N-1}x\left(n\right)W_{I}\\ \; & ≜\;\mathrm{Linear}\;\left(x,W_{R}\right)+j\cdot \mathrm{Linear}\left(x,W_{I}\right) \end{array}.$$

where x=[x(0),x(1),⋯,x(N−1)] is the chest wall displacement signal sampled from Equation (10). $W={\left[\begin{array}{cc} W_{R} & W_{I} \end{array}\right]}^{T}$ is the weight of the fully connected layer, the dimension of W is 2M×N, *M* is the number of frequencies we focus on, and *N* denotes the length of the input signal.

Given a time series x(n), its sampled values of the Z-transformation are shown in Equation (14).

(14) $$X\left(z\right)=\sum_{n=0}^{N-1}x\left(n\right)z^{-n}.$$

where sampling points $z=\frac{2\pi }{N}k,\;k=0,1,\ldots ,N-1$. However, in practical applications, only a certain segment of the entire signal spectrum is typically focused on. The emphasis is frequently placed on a specific segment of the entire signal spectrum. To enhance the accuracy of the initial frequency estimate, the chirp Z-transform (CZT) algorithm can be employed to fine-tune the spectrum in the proximity of the rough frequency estimate. This involves sampling a section of a spiral on the z-plane at equidistant angles. The sampling point is expressed as Equation (15).

(15) $$z_{k}=AW^{-k},\;k=0,1,\ldots ,M-1.$$

where $A=A_{0}e^{j\theta_{0}}$, $W=W_{0}e^{j\phi_{0}}$. Here, $A=A_{0}$ represents the radius length of the starting sampling point, $\theta_{0}$ represents the phase angle of the starting sampling point $Z_{0}$, $\phi_{0}$ represents the bisecting angle between two adjacent points, and $W_{0}$ represents the extension rate of the spiral. A value of $W_{0}$ less than 1 implies an outward extension, while $W_{0}$ greater than 1 implies an inward contraction. When $W_{0}=1$, it signifies an arc with a radius of $A=A_{0}$. If $A=A_{0}$ is set to 1, this arc becomes a segment of the unit circle. Substituting Equation (15) into Equation (14) and taking $A_{0}=1$, we obtain Equation (16).

(16) $$X\left(z_{k}\right)=\sum_{n=0}^{N-1}x\left(n\right)W_{0}^{nk}e^{-jn\left(\theta_{0}+k\phi_{0}\right)},\;k=0,1,\ldots ,M-1.$$

The number of sampling points is *M*, which is the number of frequencies of interest. Assuming the frequency range of concern is $\left[f_{1},f_{2}\right]$, the values of $\theta_{0}$ and $\phi_{0}$ can be expressed as Equation (17).

(17) $$\left\{\begin{array}{c} \theta_{0}=2\pi f_{1},\\ \phi_{0}=\frac{2\pi f_{2}}{M-1}. \end{array}\right.$$

$W_{R}$ and $W_{I}$ are equivalent to the real and imaginary parts of Equation (14), respectively. W is expressed as Equation (18).

(18) $$W_{2M,N}=\left[\begin{array}{cccc} \cos(0) & \cos\left(\theta_{0}\right) & \cdots & \cos\left(\left(N-1\right)\theta_{0}\right)\\ \cos(0) & W_{0}^{1}\cos\left[\left(\theta_{0}+\phi_{0}\right)\right] & \cdots & W_{0}^{N-1}\cos\left[\left(N-1\right)\left(\theta_{0}+\phi_{0}\right)\right]\\ \vdots & \vdots & \cdots & \vdots \\ \cos(0) & W_{0}^{M-1}\cos\left[\left(\theta_{0}+\left(M-1\right)\phi_{0}\right)\right] & \cdots & W_{0}^{\left(M-1)(N-1\right)}\cos\left[\left(N-1\right)\left(\theta_{0}+\left(M-1\right)\phi_{0}\right)\right]\\ \sin(0) & \sin\left(\theta_{0}\right) & \cdots & \sin\left(\left(N-1\right)\theta_{0}\right)\\ \sin(0) & W_{0}^{1}\sin\left[\left(\theta_{0}+\phi_{0}\right)\right] & \cdots & W_{0}^{N-1}\sin\left[\left(N-1\right)\left(\theta_{0}+\phi_{0}\right)\right]\\ \vdots & \vdots & \cdots & \vdots \\ \sin(0) & W_{0}^{M-1}\sin\left[\left(\theta_{0}+\left(M-1\right)\phi_{0}\right)\right] & \cdots & W_{0}^{\left(M-1)(N-1\right)}\sin\left[\left(N-1\right)\left(\theta_{0}+\left(M-1\right)\phi_{0}\right)\right]. \end{array}\right]$$

where $y={\left[\begin{array}{cc} y_{R} & y_{I} \end{array}\right]}^{T}$ is the output result of the fully connected layer, and the modulus operation is performed on y (as shown in Equation (19)).

(19) $$\left|y\right|=\sqrt{y_{R^{2}}+y_{I^{2}}}.$$

where $y_{R}=y\left(1:M\right)$, $y_{I}=y\left(M+1:2M\right)$.

**Figure 8:**
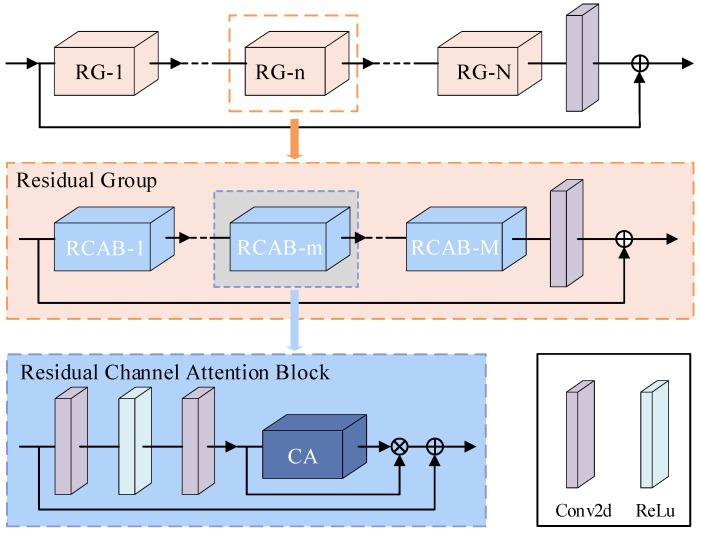
Super-resolution module. It consists of N residual groups, and each residual group contains M residual channel attention blocks.

(3) Super-resolution module: Figure 8 shows the specific structure of the super-resolution module. In order to speed up the training and improve the accuracy of network estimation, a residual in residual network [37] is used. The residual network comprises several residual groups (RG), with each group containing multiple residual channel attention blocks (RCABs). Within the RCABs, a channel attention mechanism is applied to adaptively adjust the intelligent features of each channel. It can learn the non-linear relationship between the time domain and frequency domain and extract signal features.

(4) Optimization function: For an ideal single sinusoidal signal, its amplitude spectrum manifests as a pulse signal. However, due to the limited signal length and non-unique components of the actual signal, the shape of the amplitude spectrum peak tends to resemble a Gaussian distribution. Gaussian pulses are used to generate frequency-characterized signals, and the expression is as Equation (20).

(20) $$g\left(f\right)=\alpha \cdot \exp\left(-\frac{{\left(f-f_{h}\right)}^{2}}{\sigma^{2}}\right).$$

where the frequency range is $\left[f_{1},f_{2}\right]$; here, we set it as [0, 2]. There are *M* points in the frequency domain, so the frequency accuracy is $\Delta f=\left(f_{2}-f_{1}\right)/M$. From the properties of the Gaussian function, the smaller $\sigma^{2}$ is, the more the Gaussian function tends to be an impulse function. At this time, $f_{h}$ may not be completely equal to a certain discrete scale value but is in the middle of two discrete scale values. This could cause g(f) to become a zero vector, which we want to avoid. If $\sigma^{2}$ is larger, the main lobe width will be wider and the frequency accuracy will be lower. Therefore, the values of $\sigma^{2}$ and *P* cannot be determined arbitrarily. The selection principle adopted is shown in Equation (21).

(21) 2σ=(μ+σ)−(μ−σ)=PΔf.

where the main lobe occupies *P* scales, and *P* is used to measure the frequency accuracy. α is an adjustable parameter with a value range of [1,+∞). A larger value of this parameter corresponds to an increased amplitude difference between the heartbeat frequency and other frequency components. Essentially, it signifies a higher penalty for straying from the heartbeat frequency.

The loss function is defined as the MSE function, written as Equation (22).

(22) $$L\left(f\right)=\frac{1}{N}\sum_{i=1}^{N}{\left({\hat{y}}_{i}-g_{i}\left(f\right)\right)}^{2}.$$

where $\hat{y}$ represents the frequency representation outcome of the network output. And the classic stochastic gradient descent (SGD) algorithm is used to minimize the loss function.

## 3. Experiments and Results

The radar signal utilized for detecting heartbeat frequency in this study has a duration of 4 s, with a sampling rate of 100 Hz. The relevant frequency range spans [0, 2] Hz, with 200 points, leading to the establishment of b Δf as 0.01 Hz. Assuming P=5, according to Equation (21), the value of σ can be calculated as 0.025.

HRV elucidates the inherent regularity of each heartbeat. To facilitate a comprehensive comparison and assessment of the strengths and weaknesses of various methods, this paper employs the following parameters to quantify the precision and accuracy of estimated heart rate. The average heartbeat peak-to-peak interval ($\bar{\mathrm{BBI}}$) and the standard deviation of BBI (SDBB) are calculated as Equation (23) and Equation (24).

(23) $$\bar{\mathrm{BBI}}=\frac{1}{N}\sum_{i=1}^{N}\mathrm{BBI}\left(i\right).$$

(24) $$\mathrm{SDBB}=\sqrt{\frac{1}{N}\sum_{i=1}^{N}{\left(\mathrm{BBI}\left(i\right)-\bar{\mathrm{BBI}}\right)}^{2}}.$$

where the variable *N* represents the total number of samples, and BBI(i) denotes the estimated *i*-th peak-to-peak interval, which has a reciprocal relationship with the estimated heartbeat frequency. An accurate estimation is achieved when the relative error between the estimated peak-to-peak interval and the reference peak-to-peak interval falls below a specified threshold. In this context, the permissible measurement error *d* is set at 2%. If the ratio is lower than the specified threshold *d*, it is considered that the heart rate estimation is inaccurate or the estimation error is too large. Therefore, the accuracy of heart rate detection (HRD) can be calculated as Equation (25).

(25) $$\begin{array}{cc} \; & \mathrm{HRD}=\frac{1}{N}\sum_{i=1}^{N}D\left[\frac{\left|{\mathrm{BBI}}_{\mathrm{radar}\;}-{\mathrm{BBI}}_{\mathrm{refer}\;}\right|}{{\mathrm{BBI}}_{\mathrm{refer}\;}}\leq d\right]\\ \; & D\left[x\right]=\left\{\begin{array}{cc} 1, & \;\mathrm{if}\;x\;\mathrm{is}\;\mathrm{true}\;\\ 0, & \;\mathrm{if}\;x\;\mathrm{is}\;\mathrm{flase}\; \end{array}\right. \end{array}$$

where ${\mathrm{BBI}}_{\mathrm{radar}\;}$ represents the estimated heartbeat interval, while ${\mathrm{BBI}}_{\mathrm{refer}\;}$ denotes the reference value of the heartbeat interval. Then, the heart rate detection error percentage is defined as error=1−HRD.

The average absolute error percentage (AAEP) and the average peak-to-peak interval error (APPE) serve as metrics indicating the relative disparity between the measured value and the reference value, functioning as indicators of precision. The calculations are outlined as Equations (26) and (27).

(26) $$\mathrm{AAEP}=\frac{1}{N}\sum_{i=1}^{N}\left[\frac{\left|{\mathrm{BBI}}_{\mathrm{radar}\;}\left(i\right)-{\mathrm{BBI}}_{\mathrm{refer}\;}\left(i\right)\right|}{{\mathrm{BBI}}_{\mathrm{refer}\;}\left(i\right)}\right].$$

(27) $$\mathrm{APPE}=\left|\left[\frac{1}{N}\sum_{i=1}^{N}{\mathrm{BBI}}_{\mathrm{radar}}\left(i\right)\right]-\left[\frac{1}{N}\sum_{i=1}^{N}{\mathrm{BBI}}_{\mathrm{refer}\;}\left(i\right)\right]\right|.$$

The root mean square error (RMSE) is a frequently employed indicator to quantify the deviation of observed values from their true counterparts. It is defined as Equation (28).

(28) $$\mathrm{RMSE}=\sqrt{\frac{1}{N}\sum_{i=1}^{N}{\left[{\mathrm{BBI}}_{\mathrm{radar}}\left(i\right)-{\mathrm{BBI}}_{\mathrm{refer}}\left(i\right)\right]}^{2}}$$

### 3.1. Results of Simulation Signal Frequency Estimation

The simulated respiratory signal in the dataset is derived from the respiratory model (Equation (7)) mentioned earlier, with the parameter values critical for the simulation outlined in Table 2. The respiratory frequency falls within the range of 0.12–0.48 Hz, the respiratory amplitude ranges from 4 to 12 mm, and the sampling rate is set at 100 Hz. The duration of a complete respiratory cycle is determined as the reciprocal of the respiratory frequency, that is, $T_{r}=1/f_{r}$. The inspiratory breathing time ratio is 1:1.5–1:2.5; thus, the inspiratory time length is $T_{r}=T_{r}/\left(1+r\right)$, and the exhalation time length is $T_{e}=T_{r}-T_{i}$.

**Figure 9:**
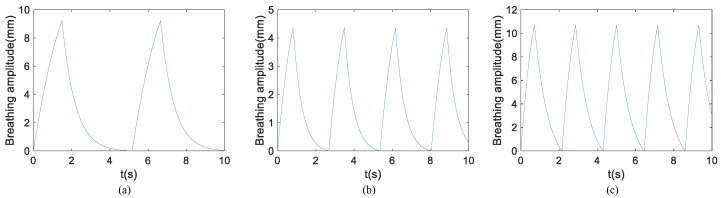
Simulated respiratory waveforms under different parameters. (**a**) $f_{r}$ is 0.194 Hz and τ is 0.69 s. (**b**) $f_{r}$ is 0.373 Hz and τ is 0.47 s. (**c**) $f_{r}$ is 0.465 Hz and τ is 0.71 s.

To emphasize the periodic nature of the respiratory signal, a 10-s time segment is selected for demonstration, although the actual data length used for training is 4 s. Within this period, qualified values for respiratory amplitude, respiratory frequency, and inhalation-to-exhalation ratio were randomly generated based on the parameter range outlined in Table 2. Subsequently, the respiratory profile time constant was determined, yielding the respiratory-induced chest wall displacement waveform presented in Figure 9. Due to the different frequency, the number of breathing cycles in 10 s is also different. In addition, different values of *r* and τ can lead to their different shapes. In Figure 9a–c, the values for $f_{r}$ are 0.194 Hz, 0.373 Hz, and 0.465 Hz, while the corresponding τ values are 0.69 s, 0.47 s, and 0.71 s, respectively.

The simulated heart signal in the dataset is derived from the heartbeat model (Equation (9)) mentioned earlier, with the parameter values critical for the simulation outlined in Table 3. The heartbeat frequency falls within the range of 0.8–1.6 Hz, and the amplitude ranges from 0.1 to 0.6 mm. The sampling rate is set as 100 Hz, and *c* is 0.1. A complete heartbeat cycle time is the reciprocal of the heartbeat frequency, i.e., $T_{h}=1/f_{h}$.

**Figure 10:**
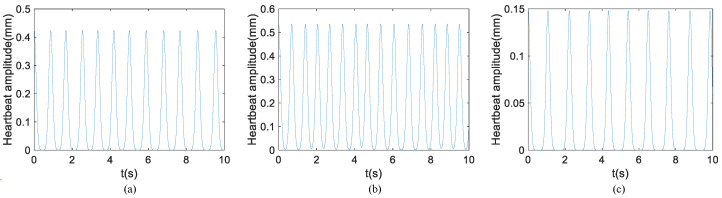
Simulated heartbeat waveforms under different parameters. (**a**) $f_{h}$ is 1.128 Hz. (**b**) $f_{h}$ is 1.493 Hz. (**c**) $f_{h}$ is 0.931 Hz.

It is known that the heartbeat time interval is a variable quantity, and by analyzing the ECG data [38] of 30 people of different genders and ages, this is indeed the case. Under the condition of normal breathing for about 10 min in a stable state, the average standard deviation of 30 people’s heartbeat interval is 46 ms. Therefore, we generate a random number from a normal distribution with a mean parameter of $T_{h}$ ms and a standard deviation parameter of 46 ms to represent a heartbeat pulse interval. The heart rate is considered to be the reciprocal of the average number of heartbeat pulse intervals over the time of observation. Figure 10 shows sample diagrams of heartbeat waveforms with different amplitudes and frequencies. In the diagram, the time interval between every two consecutive pulses is not completely equal but has a subtle gap. In Figure 10a–c, the values for $f_{h}$ are 1.128 Hz, 1.493 Hz, and 0.931 Hz, respectively.

**Figure 11:**
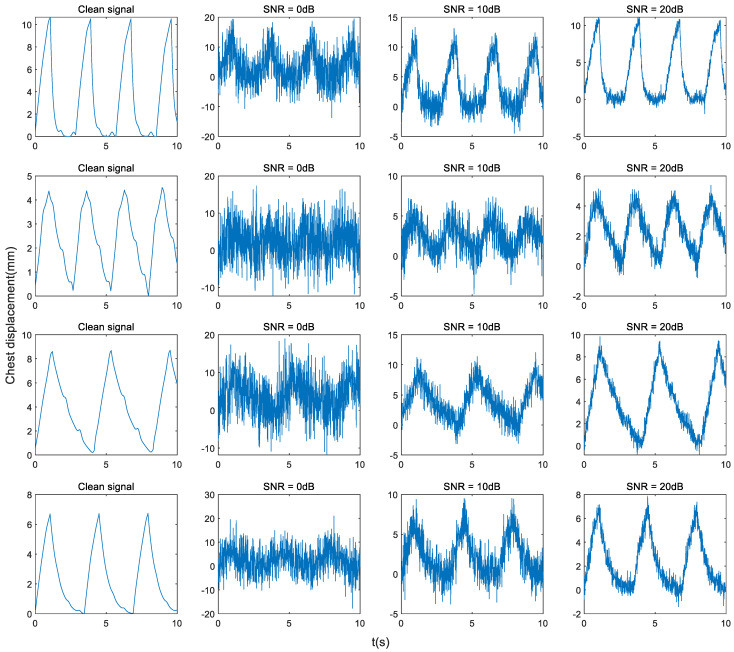
Waveforms of simulation model under different SNR and model parameters. The first columns are clean signals without noise superimposed on the breathing and heartbeat. The SNRs of the three columns on the right are 0 dB, 10 dB, and 20 dB, respectively.

Apart from respiratory and heartbeat signals, data acquired in the real collection environment may also contain other forms of noise and interference. In accordance with Equation (10), determination of chest wall displacement involves the summation of the waveforms illustrated in Figure 9 and Figure 10, as depicted in the leftmost column of Figure 11. To simulate a more realistic signal scenario, Gaussian noise is introduced to the signal generated by the simulation model. Moving from left to right, the three columns on the right represent the chest wall displacements for signal-to-noise ratios of 0 dB, 10 dB, and 20 dB, respectively. The adjacent three columns exhibit chest wall displacements corresponding to the waveform in the leftmost column in Figure 10 under SNRs of 0 dB, 10 dB, and 20 dB, respectively. Chest wall displacement waveforms were utilized for training, incorporating diverse noise levels within the SNR range of 5 to 20 dB.

**Figure 12:**
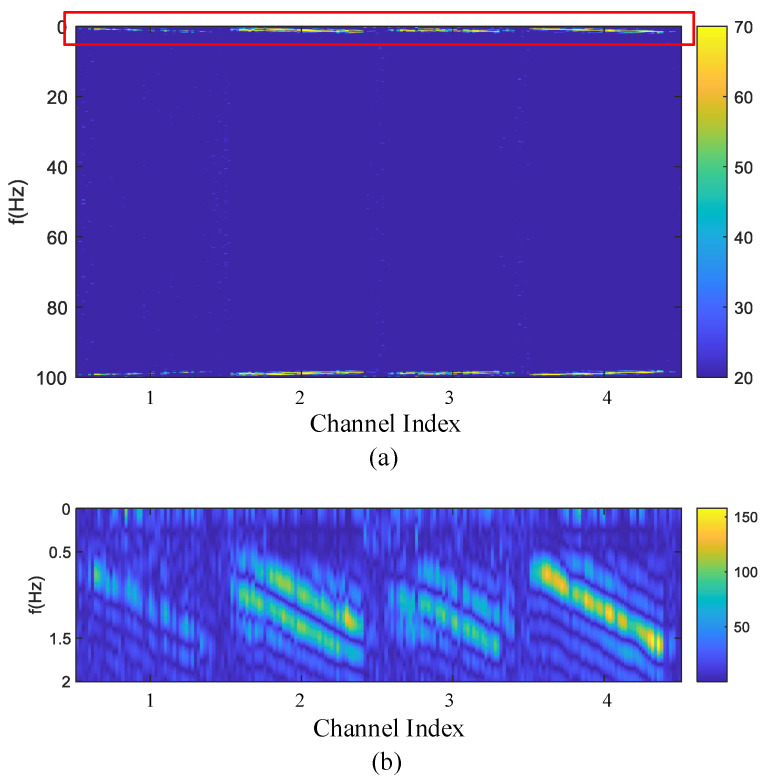
Spectrum of fully connected layer weights. (**a**) The entire frequency spectrum, including 0–100 Hz. (**b**) An enlarged version of the red-lined portion, which only includes the 0–2 Hz spectrum.

The dataset includes a training set of 30,000 samples, a validation set of 1000 samples, and a test set of 100 samples. After training, it can be seen from the spectrum of the fully connected layer weights (Figure 12) in the proposed network model that the weight spectrum of the model proposed in this paper does not occupy the entire range of the normalized spectrum. The spectrum we focus on only accounts for a small part of the normalized spectrum, and all other frequency information is ignored. This is equivalent to performing spectrum refinement to make the frequency value estimated by the model more accurate, thereby obtaining a more accurate estimate of the heartbeat frequency.

**Figure 13:**
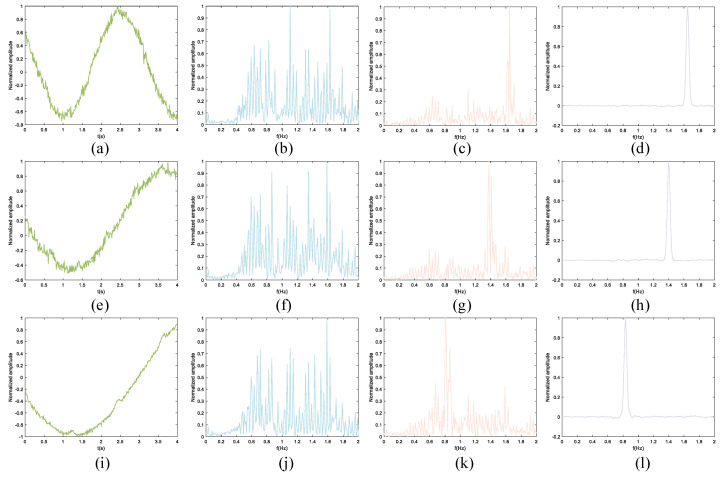
Comparison between feature maps’ output by different network layers. (**a**,**e**,**i**) Normalized input data. (**b**,**f**,**j**) Feature maps after the domain transformation module. (**c**,**g**,**k**) Feature maps after the super-resolution module. (**d**,**h**,**l**) Final frequency representations. The input signal SNR is 20 dB, and the frequency components are 1.64 Hz, 1.39 Hz, and 0.83 Hz (from top to bottom).

Figure 13 shows the feature map output by each module of the proposed network, taking an input signal with an SNR of 20 dB as an example. Figure 13a,e,i are the normalized input data, while d, h, and l are the output signals. Each row clearly presents the step-by-step process of data processing in the network. Each module undergoes a distinct transformation after specific processing, highlighting a noticeable evolution.

**Figure 14:**
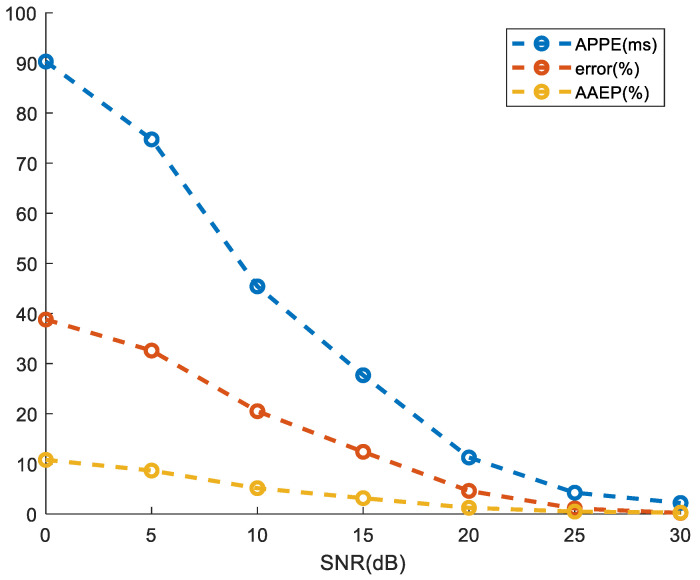
Comparison of estimation effects under different SNRs. APPE, error, and AAPE are represented by blue dotted lines, red dotted lines, and yellow dotted lines, respectively.

Figure 14 shows the results obtained for the input signal under different SNRs. Under an input signal SNR of 20 dB, the average peak-to-peak interval error is 11.27 ms, the average absolute error percentage is 1.23%, and the frequency estimation accuracy attains 95.4%. Notably, an increase in SNR corresponds to a reduction in the error associated with heartbeat frequency estimation. In scenarios where the SNR in the input signal is relatively low, both indicators, APPE and AAEP, exhibit suboptimal performance. Specifically, when the SNR is below 20 dB, APPE exceeds 20 ms, and APPE is consistently above 10%. Conversely, HRD consistently surpasses 90% under these conditions.

### 3.2. HRV Analysis Results of Two Measured Datasets

#### 3.2.1. HRV Analysis Results of FMCW Radar Data

The collection platform is described in the third part of Section II of the previous article. This experiment enlisted the participation of three individuals, with each contributing 20 min of data, resulting in a cumulative duration of 60 min. The relevant information pertaining to these individuals is outlined in Table 4. Throughout the experiment, one participant conducted four data collection sessions, each lasting 5 min. The allocation of time for the training set, validation set, and test set followed an 8:1:1 ratio, signifying that the initial 16 min of data constituted the training set, while the subsequent 4 min and last 4 min were designated as the validation set and test set, respectively. The data are cut into 4 s each. The distance between the radar and the mat was maintained at 0.7 m. Owing to variations in the body thickness of each participant, an algorithm was employed to determine the distance window for chest wall displacement, as previously mentioned.

Table 5 provides a concise summary of key metrics reflecting the performance and effectiveness of the radar in capturing and assessing heart rate data for each individual. This table presents the outcomes of the HRV analysis, providing insights into the average HR, HRD, average BBI, SDBB, RMSE, and deviations in APPE and AAEP. Notably, the data acquired through the Doppler radar exhibits a high degree of consistency with that obtained from the pulse sensor. Specifically, the average HR derived from the radar closely mirrors that extracted from the pulse sensor, with the average HR deviation remaining within a margin of 1 beat per minute. The calculated HRD for Subjects 1 and 3 both exceeded 99%, accompanied by an AAEP of less than 0.5%. This outcome underscores the comparability between the heart rate results derived from radar data and those obtained from the pulse sensor. The demonstrated success of our proposed algorithm suggests its potential utility as a tool for HRV analysis.

**Figure 15:**
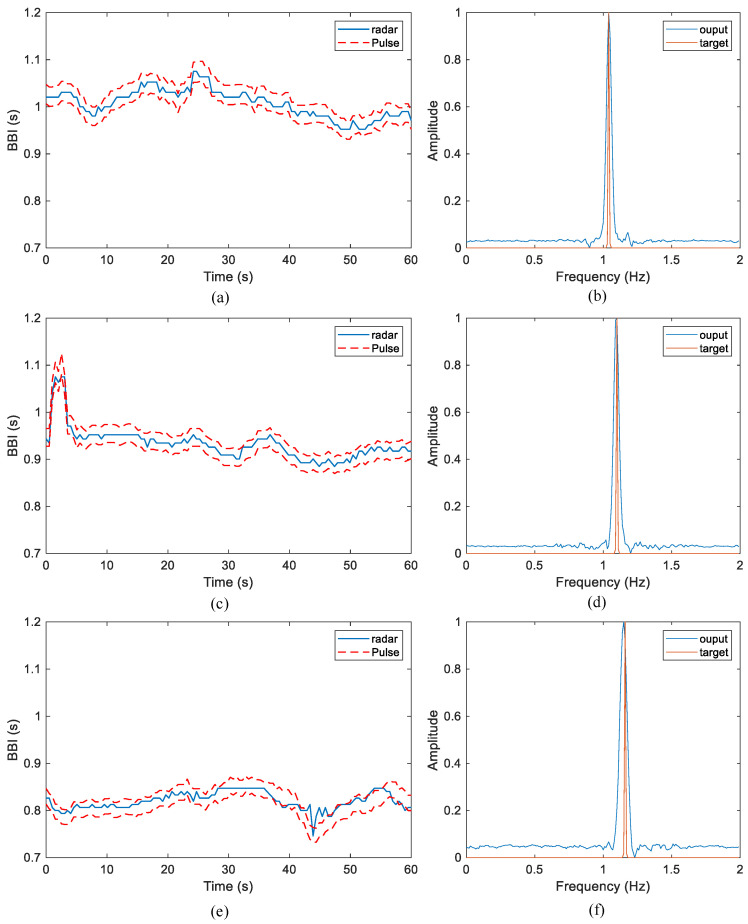
HRV monitoring results. (**a**,**c**,**e**) are the heartbeat peak-to-peak interval tracking results of three people’s one-minute data, respectively. (**b**,**d**,**f**), respectively, show the comparison between the 30 s data network output results of three people and the reference spectrum.

Figure 15 illustrates the HR monitoring outcomes generated by the proposed algorithm. The red dotted lines depicted in Figure 15 represent the two boundaries that define ±2% deviation around the reference BBI, as per Equation (24). These boundaries serve as a metric to evaluate the monitoring performance. Concerning the parameter α in Equation (20), guided by the principles outlined in Equation (21), it was set to 20 for this experiment. To facilitate a more convenient comparative analysis between the network output spectrum and the reference spectrum, normalization is applied to the network output results. The comparison of radar and ECG-recorded heart rates is conducted through the detection of spectral peaks. Notably, the one-minute dataset presented in the figure consistently falls within the allowable error range, i.e., within the reference boundary. This substantiates the algorithm’s proposed high accuracy in tracking heart rate.

**Figure 16:**
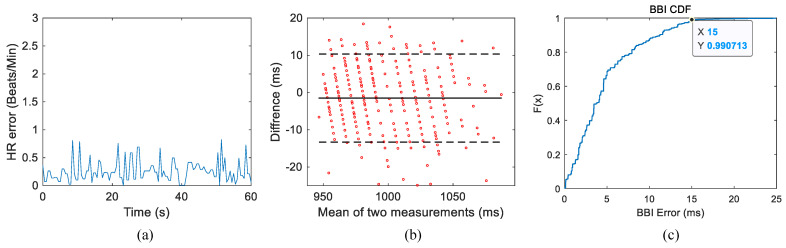
(**a**) Deviation of BBI from its reference value. (**b**) Bland–Altman plot of BBI. (**c**) Cumulative distribution function of BBI error.

Figure 16 presents the heart rate estimation outcomes for Subject 1, intended for comparison with the reference true value of heart rate. In Figure 16a, it is evident that the deviation of HR from its reference value is less than one beat per minute. Figure 16b illustrates the alignment between the reference true value acquired through ECG and the estimation results derived from BBI radar data. The central solid line represents the mean difference between the two measurement methods, while the upper and lower dotted lines depict ±1.96 times the standard deviation of the difference. The scatter points are uniformly distributed within the standard deviation lines, with the mean line approximating 0. This indicates a relatively close proximity between the two measurement results, signifying good consistency. The abscissa of Figure 16c is the difference in BBI obtained by the two measurement methods. The graph reveals that data with an error exceeding 15 ms do not surpass 1%, and the majority of heart rate estimation results fall within the measurement error threshold.

#### 3.2.2. HRV Analysis Results of CW Radar Data

Schellenberger et al. presented a radar vital signs dataset [38] utilizing a radar carrier frequency of 24 GHz. The radar is directed towards the chest, concurrently collecting electrocardiogram, impedance cardiogram, and non-invasive continuous blood pressure data. In this experiment, the electrocardiogram frequency serves as the reference value. The dataset encompasses various signals recorded from 30 individuals in five distinct states, including the Valsalva maneuver, holding breath, and the tilt table test. There are 14 male and 16 female healthy subjects, with an average age of 26.6 ± 4.0 years for men and 34.3 ± 11.9 years for women.

Initial data preprocessing involves extracting phase information from the radar I and Q channel data, followed by filtering and downsampling to standardize the sampling rate to 100 Hz. Subsequently, data segmentation occurs, with each segment comprising 400 points, equivalent to 4 s, and utilizing a sliding window of length 0.05 s. As previously performed, the dataset is partitioned in an 8:1:1 ratio, and the model is retrained. The obtained results are presented in Table 6.

The average heart rate for each individual exhibits a negligible discrepancy of no more than one beat per minute, with the maximum difference recorded at 0.819 beats per minute. Additionally, the average peak-to-peak intervals demonstrate variations of less than 15 ms, with the most significant difference being 14.081 ms. SDBB and RMSE were employed to evaluate the general characteristics of HRV. The average differences in SDBB and RMSE between radar and ECG measurements are 5.46 ms and 22.23 ms, respectively. In a medical context, the relative error percentage for BBI remains within 2%, establishing the accuracy of heart rate estimation. The average measurement accuracy across 30 individuals exceeds 95%. Moreover, the average values for AAEP and APPE among the 30 subjects are 0.86% and 2.22 ms, respectively. Notably, Subject No. 18 exhibits the highest average heart rate, BBI estimation error, and APPE, while Subject 25 demonstrates the most considerable SDBB deviation between the two measurement methods.

**Figure 17:**
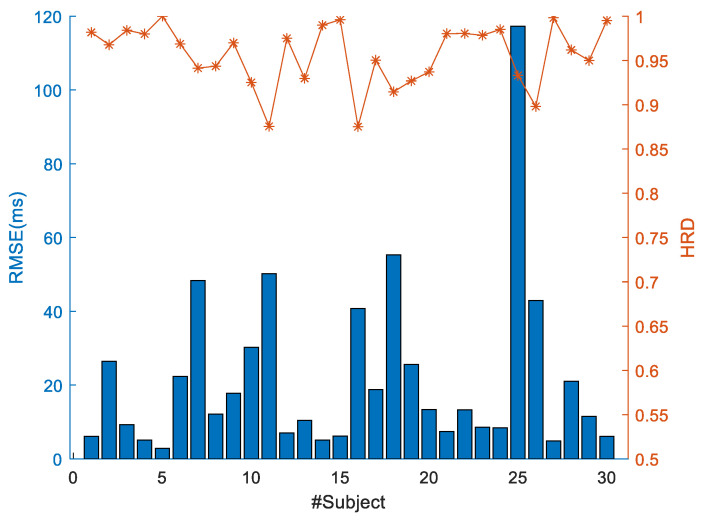
RMSE of BBI estimation and HRD for 30 subjects.

Figure 17 provides a more intuitive visualization of two indicators, namely RMSE and HRD. The majority of RMSE values are below 20 ms, with accuracy rates surpassing 80%, indicating a concentrated estimated success rate around 95%.

### 3.3. Performance Comparison of Different Methods

**Figure 18:**
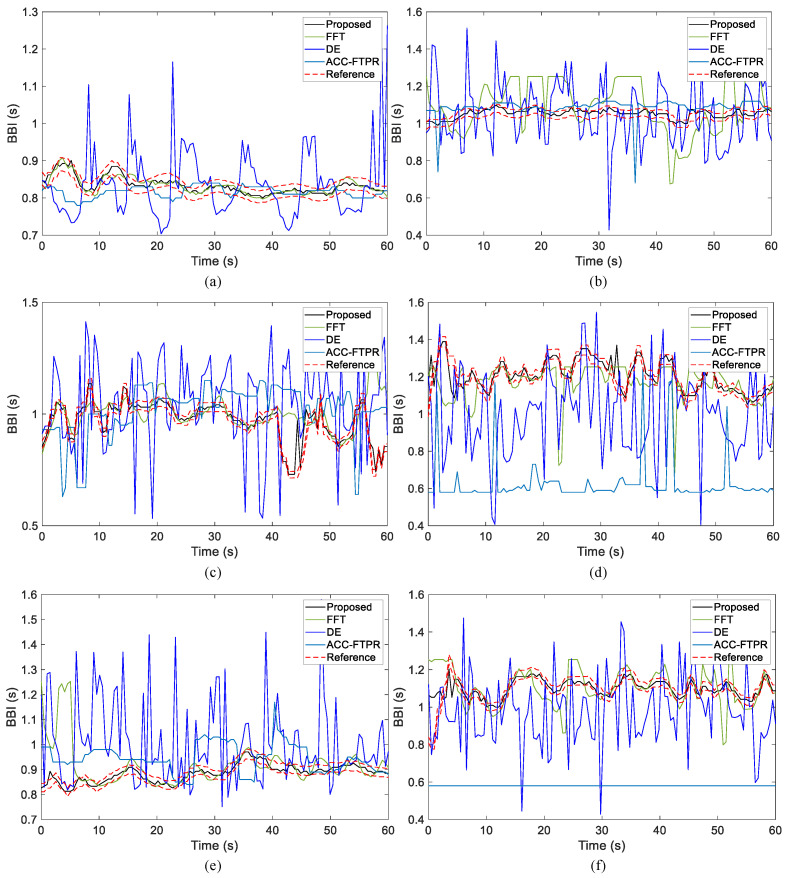
Comparison of HRV estimation results of different methods among 6 subjects. (**a**–**f**) are comparisons of results obtained by different methods for the data of subjects 1, 6, 11, 16, 21 and 26, respectively.

To highlight the advantages of the proposed method, a comparative analysis is conducted against the FFT, DE [25], ACC-FTPR [18], and HeRe [27] methods. In Figure 18a–f, a detailed comparison of heartbeat detection results is presented for the proposed method and the aforementioned three methods across Subjects 1, 6, 11, 16, 21, and 26. Each figure illustrates continuous heartbeat monitoring results over one minute, with the black line representing the predicted heartbeat results from our method and the red dashed line depicting reference heartbeat results obtained by the ECG sensor. The results from the other three methods are represented by distinct colors. Notably, the estimated results from our method align closely with the reference values, demonstrating consistency and adaptability to changes in the reference values. Conversely, the accuracy of estimation results from other methods is comparatively lower, and their stability is notably reduced. In general, the estimation results from these alternative methods are not as reliable as those obtained using the proposed approach in this article.

To facilitate comparison, a success rate is defined in Equation (29).

(29) $$P=\frac{1}{N}\sum_{i=1}^{N}D\left[\left|{\mathrm{HR}}_{\mathrm{radar}\;}\left(i\right)-{\mathrm{HR}}_{\mathrm{refer}\;}\left(i\right)\right|\leq \Delta F\right]$$

where ΔF is set to 4 BPM. Table 7 presents a comprehensive comparison of the proposed HRV monitoring method with four alternative methods, showcasing the excellent performance of the proposed approach across various evaluation indicators. The DE method and ACC-FFT method exhibit suboptimal performance on HRD indicators, primarily due to their measurement accuracy exceeding the acceptable error range. Consequently, the performance of these two methods on the success rate *P* indicator is also unsatisfactory for the same reason. Moreover, the FFT method lags behind the proposed method in terms of stability, as evidenced by an RMSE value nearly six times higher than that of the proposed method. This contrast is further highlighted in Figure 18b.

## 4. Discussion

This paper employs the arctangent demodulation method [14] to extract the phase information for deriving the chest wall displacement of the target. Subsequently, a band-pass filter is applied to temporarily eliminate the respiratory signal. The proposed network is then deployed to further suppress breathing harmonics. As depicted in Figure 18 and summarized in Table 7, comparative analysis reveals that the method proposed in this article exhibits superior accuracy in eliminating respiratory harmonics compared to conventional techniques such as FFT or DE [25]. Notably, the ACC-FTPR [18] demonstrates suboptimal performance when employing a short data acquisition window (4 s) for analysis.

While the proposed method demonstrates effective heart rate variability monitoring in static scenes, motion artifacts present a significant challenge in sports scenes, compromising heart rate estimation accuracy. Target movement alters the distance between the radar and the subject, inducing variations in echo signal phase and amplitude, thereby impeding heart rate signal extraction. To address this issue, motion compensation algorithms can be explored, involving preprocessing steps such as estimating target motion trajectory and velocity to mitigate interference. Additionally, deep learning techniques offer potential solutions, as exemplified by Professor Gu’s deep neural network-based body motion elimination technology [39] and C. Ye et al.’s deep clustering method [40,41].

Furthermore, the field encounters obstacles in multi-target recognition and separation. Radar detection may encompass multiple targets, causing echo signals to interfere and complicating the extraction of individual heart rate information. Approaches to this challenge include signal processing for feature extraction, leveraging radar spatial resolution for intelligent spatiotemporal fusion [29], and employing machine learning or deep learning algorithms [42] for automated multi-target identification and separation.

In contexts ranging from medical emergencies to sports training and daily health monitoring, rapid access to heart rate information holds paramount importance. Our proposed method, requiring only 4 s of data, enables frequent heart rate monitoring and accurately captures subtle variability, thus facilitating cardiac function assessment and disease risk prediction. However, the majority of existing methods rely on laboratory health data, necessitating an evaluation of their applicability in real-world scenarios. Factors such as diverse demographic groups and individual variability may impact algorithmic accuracy. Through comprehensive data testing, the algorithm’s stability and accuracy can be assessed, delineating its scope of application and limitations.

## 5. Conclusions

This article introduces a novel deep learning-based methodology employing Doppler radar for the extraction of human heartbeat information. The proposed network demonstrates proficiency in providing accurate assessments of human HR and precise monitoring of HRV through the utilization of brief 4-second radar signals. The method’s efficacy was validated using both simulated signals and authentic data, with comparative analyses against several alternative methodologies. The results highlight the heightened accuracy and robustness of our proposed method in HRV estimation. On the FMCW radar dataset, the Bland–Altman index indicates a negligible bias in our method, with the majority of BBI errors falling below a specified threshold. Additionally, in the CW radar dataset involving 30 subjects, our method achieved an average accuracy surpassing 95%.

## Figures and Tables

**Table 1:** Radar sensor parameter settings. It mainly includes some information about the parameters of the FMCW signal emitted by the radar.

Parameter Name	Value
Start Frequency	77 GHz
Frequency Slope	80 MHz/μs
Chirp Circle Time	58 μs
Idle Time	8 μs
Ramp End Time	50 μs
Frame Periodicity	5 ms
ADC Samples	200
Number of Chirp Loops (*M*)	5
Number of Frames (*N*)	60,000

**Table 2:** Breathing model simulation parameters.

Parameter Name	Value
$A_{r}$	4–12 mm
$f_{r}$	0.12–0.48 s
$T_{r}$	2.08–8.33 s
*r*	1:1.5–1:2.5 s
τ	0–$T_{r}$ μs

**Table 3:** Heartbeat model simulation parameters.

Parameter Name	Value
$A_{h}$	0.1–0.6 mm
$f_{h}$	0.8–1.6 s
$T_{h}$	0.625–1.25 s

**Table 4:** Overview of all test subjects.

ID	Age	Gender	Height	Weight
1	23	Female	164	45
2	31	Male	165	55
3	27	Female	168	52

**Table 5:** Average HR and evaluation indices obtained via FMCW radar for three subjects.

Subject	Avg.HR (BPM)		Avg.BBI (ms)		SDBB (ms)		RMSE	AAEP	APPE	HRD
	Radar	ECG	Radar	ECG	Radar	ECG	(ms)	(%)	(ms)	(%)
1	65.06	65.08	923.04	922.78	27.43	27.94	4.46	0.36	1.48	99.87
2	71.14	70.94	845.34	848.09	39.99	43.28	12.78	1.16	0.26	85.64
3	60.12	60.03	998.84	1000.30	28.70	29.50	6.22	0.48	2.83	99.66

**Table 6:** Average HR and evaluation indices obtained via Doppler radar for 30 subjects.

Subject	Avg.HR (BPM)		Avg.BBI (ms)		SDBB (ms)		RMSE	AAEP	APPE	HRD
	Radar	ECG	Radar	ECG	Radar	ECG	(ms)	(%)	(ms)	(%)
1	71.86	71.88	835.52	835.26	22.15	21.66	6.12	0.54	0.26	98.18
2	63.37	63.22	949.40	950.87	47.71	42.07	26.46	0.86	1.47	96.77
3	62.79	62.80	960.77	960.78	71.06	72.10	9.29	0.63	0.01	98.41
4	73.18	73.35	820.70	818.90	26.53	27.39	5.10	0.44	1.80	98.00
5	62.53	62.56	962.68	962.14	54.94	54.52	2.85	0.24	0.54	100.00
6	56.94	57.24	1054.40	1048.60	28.78	19.94	22.37	0.83	5.75	96.87
7	58.40	58.01	1043.50	1049.90	131.54	130.23	48.34	1.25	6.39	94.14
8	56.12	56.11	1072.30	1072.40	55.65	55.18	17.82	0.91	0.12	92.13
9	60.16	60.18	1003.50	1003.10	79.45	79.26	17.80	0.70	0.38	96.98
10	45.43	45.55	1329.00	1326.60	99.80	104.52	30.24	1.04	2.41	92.52
11	61.62	62.38	981.65	971.76	84.36	93.35	50.18	2.13	9.88	87.56
12	65.40	65.50	919.35	918.02	42.61	43.07	7.04	0.54	0.61	97.51
13	66.29	66.41	905.72	904.15	23.35	24.15	10.43	0.87	1.57	92.98
14	80.03	80.01	750.60	750.73	25.64	25.40	5.13	0.42	0.12	98.98
15	63.55	63.57	947.26	947.04	53.55	53.90	6.20	0.50	0.59	99.60
16	50.08	50.11	1203.50	1202.50	80.72	78.70	40.78	1.47	1.01	87.52
17	59.22	59.57	1014.60	1009.10	38.30	42.90	18.81	0.94	5.42	95.04
18	65.92	65.10	910.99	925.07	27.19	60.53	55.29	1.75	14.08	91.46
19	48.52	48.54	1239.30	1239.00	56.58	59.41	25.58	1.02	0.29	92.69
20	60.41	60.32	996.76	998.17	57.67	58.41	13.41	0.86	1.42	93.71
21	67.70	67.59	887.55	888.89	33.34	32.56	7.45	0.63	1.34	98.04
22	54.74	54.70	1096.70	1097.60	25.66	27.03	13.32	0.55	0.84	98.05
23	55.20	55.20	1089.10	1089.10	48.94	48.74	8.57	0.57	0.05	97.85
24	69.43	69.48	865.53	864.92	34.95	35.30	8.40	0.48	0.61	98.50
25	66.38	65.91	910.32	913.38	122.41	54.65	117.25	1.79	3.06	93.32
26	54.83	55.00	1096.90	1095.50	53.20	67.30	49.14	1.62	1.41	91.06
27	59.06	59.05	1016.50	1016.70	23.63	23.53	4.90	0.38	0.20	99.82
28	53.20	53.31	1133.00	1130.70	74.34	74.89	21.04	0.80	2.34	96.19
29	63.33	63.48	950.45	948.27	54.70	54.95	11.51	0.79	2.18	95.00
30	50.99	51.00	1185.40	1185.00	1.36	1.36	6.09	0.32	0.38	99.53

**Table 7:** Performance comparison of different methods corresponding to long-term continuous monitoring results.

Methods	RMSE	AAEP	APPE	HRD	P
DE	195.16	16.29	148.04	5.5	43.06
FFT	118.81	7.48	46.48	38.14	74.07
ACC-FTPR	229.21	17.45	91.03	8.44	26.58
HeRe	-	-	-	-	95.88
**Proposed**	**21.83**	**0.86**	**2.24**	**95.65**	**98.53**

## Data Availability

The data presented in this study are available on request from the corresponding author due to privacy.
